# Susceptible-Infected-Susceptible type COVID-19 spread with collective effects

**DOI:** 10.1038/s41598-023-49949-7

**Published:** 2023-12-18

**Authors:** Amanda Crocker, Daniel Strömbom

**Affiliations:** Department of Biology, Lafayette College, Easton, PA 18042 USA

**Keywords:** Infectious diseases, Applied mathematics, Infectious diseases, Human behaviour

## Abstract

Many models developed to forecast and attempt to understand the COVID-19 pandemic are highly complex, and few take collective behavior into account. As the pandemic progressed individual recurrent infection was observed and simpler susceptible-infected type models were introduced. However, these do not include mechanisms to model collective behavior. Here, we introduce an extension of the SIS model that accounts for collective behavior and show that it has four equilibria. Two of the equilibria are the standard SIS model equilibria, a third is always unstable, and a fourth where collective behavior and infection prevalence interact to produce either node-like or oscillatory dynamics. We then parameterized the model using estimates of the transmission and recovery rates for COVID-19 and present phase diagrams for fixed recovery rate and free transmission rate, and both rates fixed. We observe that regions of oscillatory dynamics exist in both cases and that the collective behavior parameter regulates their extent. Finally, we show that the system exhibits hysteresis when the collective behavior parameter varies over time. This model provides a minimal framework for explaining oscillatory phenomena such as recurring waves of infection and hysteresis effects observed in COVID-19, and other SIS-type epidemics, in terms of collective behavior.

## Introduction

Since the twentieth century, the world has seen multiple outbreaks of infectious diseases, including the Spanish flu, HIV/AIDS, SARS, Swine flu, Ebola, Zika, and most recently COVID-19^1^. Transmission of the severe acute respiratory syndrome coronavirus 2 (SARS-CoV-2) was identified as the root cause of Coronavirus disease 2019 (COVID-19) in January 2020^2^. By March 2020, the World Health Organization (WHO) classified COVID-19 as a global pandemic, and containment efforts to stop the spread of the virus began soon after. Early 2020 estimates for the transmissibility of the original virus suggested that each infected individual would spread the virus to 1.4–2.5 people on average^3^. Since then, the infectiousness of the virus has intensified, with an estimated 9.5 people becoming infected from each person with the Omicron variant of 2021^4^. Over three years later, COVID-19 continues to persist in society, notably in recurring waves of infection^5–7^ that are influenced by changes in compliance with mitigation efforts and guidelines^6,8,9^.

Mitigation efforts are thought to help curb infection spread. A 2022 CDC report found that individuals who wore face masks were at least 56% less likely to test positive for COVID-19, in conjunction with any other protective measures they were taking^10^. Social distancing also contributes to limiting COVID-19 spread, as the ten countries with the most COVID-19 cases in early 2020 saw a decline in active cases within four weeks of initiating social distancing^11^. To make these protective changes in physical behavior, individuals must have received effective communication about the current status of infection in order to personally weigh the risks associated with certain actions^12–14^. Thus behavior is also linked to media representation and portrayal of the pandemic’s status locally, nationally, and globally^15–17^. While each individual must make their own decisions regarding how to behave in a pandemic environment, the collective decisions of an entire population will determine the fate of infection spread throughout a community^18^. Conversely, the degree of infection spread and the perceived threat of disease also contribute to the willingness of the population to follow recommendations and precautions^12^. A study of the 1918–1919 Influenza A outbreak found that as the severity of the epidemic increased, there were correspondingly high levels of perceived risk and increased indications that policy played a role in forming public perceptions. These three factors fluctuated together as the epidemic incidence became more and less severe, indicating that willingness to cooperate with mitigation recommendations contributes to infection dynamics and vice versa^19^. Ultimately, even with government or societal recommendations, the willingness and ability of a population to follow these recommendations as a community drives whether or not these guidelines are effective^20^.

Modeling disease spread may contribute to uncovering what factors affect the dynamics of infectious diseases and be used for forecasting^21^. For example, a model may be used to estimate the expected number of cases within a specific time period or the anticipated impacts of different mitigation strategies to support public health decision-making^22^. A large number of models have been developed to forecast and investigate different aspects of the COVID-19 pandemic. In particular, so-called compartmental models where the population is partitioned into a number of compartments, e.g. susceptible and infected, and rates for the flow between the different compartments are specified, have a very long history in disease spread modeling^23–36^. The SIR (susceptible, infected, recovered) model^37^ has been particularly important to the study of infectious diseases, and has served as a base model for many COVID-19 model variations and extensions, for example, the SEIR^28–30,38^, SAIU^31^, SAIR^32^, SIRSi^25^, SLIAR^34^, SEIRD^33^, SEAHIR^23,35^, and SIDARTHE^36^ models. Agent-based models where individual-level behaviors and interactions are specified and the community-level spread that emerges from those individual-level features studied via simulation have also been used extensively to model COVID-19^39–43^.

Collective behavior, especially collective cooperation or defection, has been incorporated into a number of models^44–47^. However, many of these models are highly complex due to their large number of states/compartments (SIR+) and parameters, which limits their use as tools to investigate causal links between specific factors and specific features of the spread. As the COVID-19 pandemic progressed it was observed that individual reinfection was common^48,49^. Calling the inclusion of the recovered class in compartmental models into question, an SI model was constructed by generating an exponential fit to early COVID-19 data^50^. However, this model does not account for collective behavior, and we cannot find any other SI or SIS models for COVID-19 in the literature that investigates the impacts of collective behavior. This is unfortunate, given that individual reinfection is an empirical fact and that collective behavior is known, or suspected, to affect and drive certain features of the spread. For example, that the reinfection waves are influenced by collective changes in compliance with mitigation efforts and guidelines^6,8,9^. While collective behavior plays a role in other SIS type epidemics, e.g. influenza, the collective mitigation efforts in response to COVID-19 are unparalleled by recent epidemics and therefore an ideal starting point for modeling efforts aimed at developing an understanding of collective behavior in SIS-type epidemics.

## Model and results

**Figure 1:**
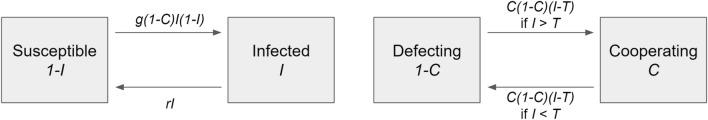
Schematic representation of the extended SIS model with collective effects. $I=I(t)$ is the proportion of population infected at time *t*, $C=C(t)$ is the proportion of the population cooperating, *g* is the disease transmission rate, *r* is the disease recovery rate. The left diagram illustrates the flow between susceptible and infected and is identical to the standard SIS model, except that the constant transmission rate *g* in the SIS model has been replaced by a cooperation-dependent transmission rate $g(1-C)$, and *C* is governed by the process in the right diagram. In particular, *C* decreases in a logistic type manner if the disease prevalence *I* is less than the collective cooperation threshold *T*, and *C* increases if *I* is larger than *T*.

Here we extend the standard SIS model^21,50^ to include collective behavior. See Fig. 1. The proposed general model becomes a set of two ordinary differential equations, one for the proportion of infected ($I=I(t)$) and one for the proportion of cooperators ($C=C(t)$) of the form

1 $$\begin{aligned} {\left\{ \begin{array}{ll} \displaystyle {\dot{I}}=g(1-C)I(1-I)-rI \\ {\dot{C}}=C(1-C)(I-T) \end{array}\right. } \end{aligned}$$

where *g* is the disease transmission rate and *r* is the recovery rate from the standard SIS model, and *T* is a collective cooperation threshold described below. Note that the top equation generalizes the SIS model in that a cooperation-dependent disease transmission rate $g(1-C)$ replaces the constant disease transmission rate *g* used in the standard SIS model. This factor was defined in this way to make disease transmission 0 when every individual is cooperating ($C=1$) and makes the model reduce to the standard SIS model when there is no cooperation ($C=0$). The bottom equation which governs the evolution of cooperative behavior was chosen to be a logistic-type equation with an additional factor that regulates whether cooperation is increasing or decreasing depending on whether the infection prevalence *I* in the community is above or below the threshold *T*. The rationale for the logistic part of the equation, i.e. ${\dot{C}}=C(1-C)$, is that it is the simplest model of a diffusion type process. It has an s-shaped solution that mimics common behavioral acquisition patterns observed in imitation processes in animal^51^ and human^52^ communities. The additional factor $(I-T)$ is included to model the empirical observation that infection prevalence influences the willingness of a population to cooperate and follow recommendations^12^. The threshold *T* corresponds to the sensitivity of the community to disease prevalence. A low threshold will lead to cooperative behavior even at low infection prevalence, and a high threshold will require a higher infection prevalence before the community starts to cooperate with safety recommendations and guidelines.

### Qualitative analysis of the general model

We employed standard qualitative analysis for 2D ODE systems to obtain the following main result.

The four equilibria of the model and their stability properties $\forall (g,r,T)\in (0,1)^3$ with $g\ne r$ are

1. $I=0$ and $C=0$ is a stable node if $g<r$ ($\forall T$).
2. $I=0$ and $C=1$ is always unstable.
3. $I=1-\frac{r}{g}$ and $C=0$ is a stable node if $r<g<\Delta$ ($\forall T$).
4. $$I=T$$ and $$C=1+\frac{r}{g(T-1)}$$ is a

stable spiral if $r<A$ and $g>\Gamma$

stable node if $r<A$ and $\Delta<g<\Gamma$

stable node if $r>A$ and $g>\Delta$

where $$A=\frac{4(T-1)^2}{T}$$, $$\Gamma =\frac{4r(1-T)}{4(T-1)^2-rT}$$, and $$\Delta =\frac{r}{1-T}$$.

See the “Methods and calculations” section for the derivation of this result.

### COVID-19 parameterization of the general model

For some analyses of the model (1), estimates of *r* and *g* specific to COVID-19 are required and we use the following estimates. The standard conservative incubation period for COVID-19 is up to 14 days^53^, so we set the recovery rate to $r= 1/14$, as the probability of recovering on any one time step. For the transmission rate, *g*, we used the estimated basic reproductive number $R_0$ for the ancestral strain of SARS-CoV-2 of 2.9^54^, the relationship $R_0 = gr$^21^, and $r= 1/14$, to obtain the estimate $g = 0.2$.

#### Classification of the equilibria in the model with COVID-19 recovery rate $r=1/14$

**Figure 2:**
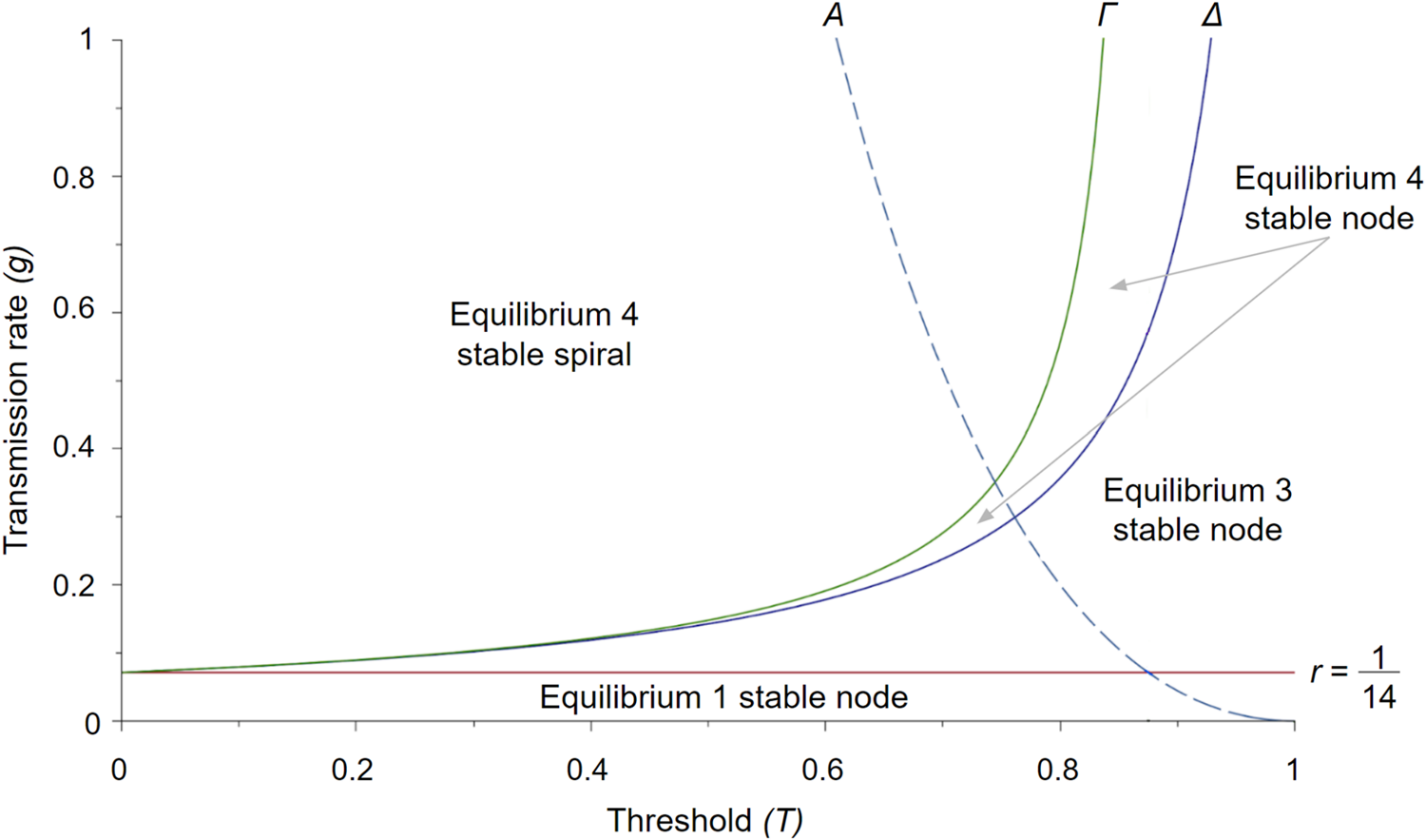
Type of equilibria for constant recovery rate. With fixed recovery rate $r = 1/14$ the long-term stability properties of the system can be illustrated in the (*g*, *T*)-plane. There are four possible outcomes determined by where the parameter pair (*g*, *T*) of interest is located relative to the four curves $r=1/14$, *A*, $\Gamma$, and $\Delta$. For $g<\Delta$ the two standard SIS model equilibria are stable $g<r$. More specifically, for $r<g$ equilibrium 1 $(I,C)=(0,0)$ is a stable node, if $r<g<\Delta$ equilibrium 3 $(1-r/g,0)$ is a stable node. For $g>\Delta$ equilibrium 4 $(T,1+r/g(T-1))$ is stable. A stable node if $\Delta<g<\Gamma$, and a stable spiral if $g>\Gamma$.

When the recovery rate is fixed to $r=1/14$, how the equilibria and their stability properties depend on the parameters *g* and *T* can be illustrated in a phase diagram. Figure 2 depicts the *A*, $\Gamma$, and $\Delta$ curves as functions of *g* and *T* and indicates the stability of the equilibria present in each region. For fixed $g>1/14$ we note that as *T* increases from a low value, the stability of equilibrium 4 changes from a stable spiral to a stable node when it reaches the $\Gamma$ curve, and then when it reaches the $\Delta$ curve equilibrium 4 becomes unstable and equilibrium 3 becomes a stable node.

#### Classification of the equilibria in the model with COVID-19 recovery rate $r = 1/14$ and transmission rate $g = 0.2$

**Figure 3:**
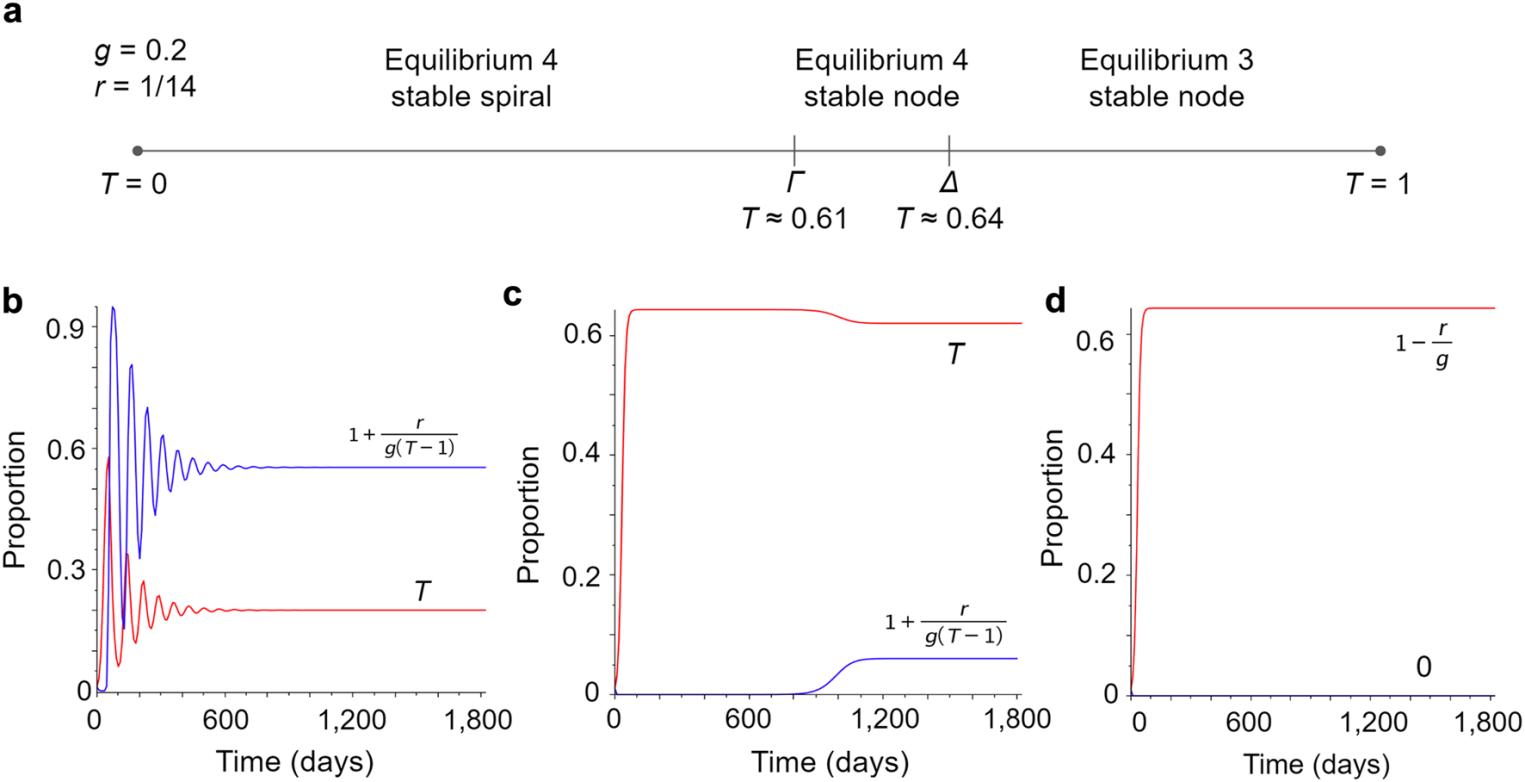
Type of the equilibria for constant transmission and recovery rates. For the ancestral COVID-19 parameters $r=1/14$ and $g=0.2$, the possible stability outcomes depend only on the value of *T*. (**a**) Line indicating which values of *T* result in the three outcomes in terms of the intersections with the $\Gamma$ and $\Delta$ curves. (**b**–**d**) Shows the changes in proportions infected (red) and cooperating (blue) over five years time for different values of *T*. (**b**) Proportion infected and cooperating curves for $T=0.2$. For any value of $T < 0.61$ we expect the proportions infected and cooperating to exhibit damped oscillations while converging to equilibrium 4 $(T,1+r/g(T-1))$, and with the parameters used here that would be (0.2, 0.55) which is reflected in the figure. (**c**) Proportion infected and cooperating curves for $T=0.62$. Because *T* satisfies $0.61< T < 0.64$ we expect the proportions infected and cooperating to approach equilibrium 4 $(T,1+r/g(T-1))$, here (0.62, 0.06), which is reflected in the figure. We note that initially the infection grows rapidly towards the standard SIS model equilibrium ($1-r/g=0.64$) and only when it passes $T=0.62$ does cooperation kick in and eventually drags the infection down to 0.62 where it stabilizes. (**d**) Proportion infected and cooperating curves for $T=0.7$. For any value of $T > 0.64$ equilibrium 3 $(1-r/g,0)$ is stable, i.e. the proportion infected *I* will approach the standard SI model equilibrium $1-r/g=0.64$ and cooperation *C* will approach 0. We see this reflected in the figure. This is a consequence of the fact that the threshold *T* is larger than the standard SIS model equilibrium $1-r/g$, so *I* will never exceed *T*, and therefore cooperation will decrease towards 0.

When the disease transmission rate is fixed to $g = 0.2$ and the recovery rate to $r = 1/14$, the value of the threshold *T* alone dictates which equilibrium is stable and its stability properties. Figure 3a illustrates the outcomes on a phase line for *T*. We note that for increasing *T* the transition from the stable spiral to the stable node for equilibrium 4 occurs at the intersection with the $\Gamma$ curve, where $T \approx 0.61$, and the transition from equilibrium 4 being a stable node to equilibrium 3 being a stable node occurs at the intersection with the $\Delta$ curve, where $T \approx 0.64$. Figure 3b shows the infection and cooperation curves for $T = 0.2$, which corresponds to equilibrium 4 being a stable spiral. Note that Fig. 3b indicates that spikes in cases will occur roughly every 100 days. Figure 3c shows infection and cooperation curves when $T = 0.62$, which corresponds to equilibrium 4 being a stable node. Figure 3d shows infection and cooperation curves when $T = 0.7$, which corresponds to equilibrium 3 being a stable node.

#### Hysteresis behavior in the model with COVID-19 recovery rate $r = 1/14$ and transmission rate $g = 0.2$

**Figure 4:**
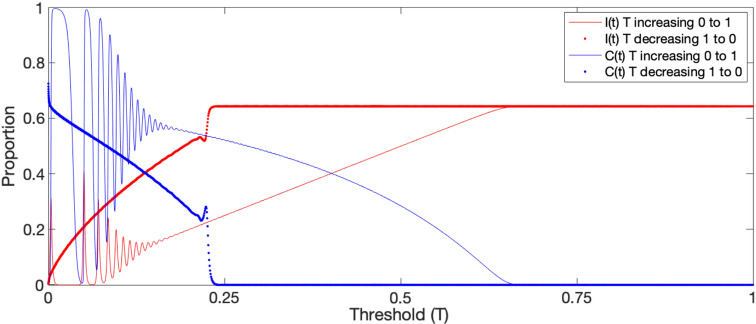
Hysteresis effect with varying collective cooperation threshold *T*. As *T* increases from 0 to 1 the proportion of infected *I* and cooperating *C* initially exhibit damped oscillations as in Fig. 3b, but instead of stabilizing at equilibrium 4, as it would for a fixed *T*, the increasing threshold eventually renders equilibrium 4 unstable and the system instead approaches and stabilizes at equilibrium 3 ($C=0$ and $I=1-r/g$). From there, when *T* decreases from 1 back to 0, the proportion infected and cooperating does not follow the increasing *T* path in reverse. Instead, the system remains in equilibrium 3 until *T* has decreased beyond 0.25 at which point the system abruptly switches to approach equilibrium 4 and bypasses the oscillatory phase observed when *T* was increasing. Indicating that this system exhibits substantial hysteresis effects.

We also investigated the impacts of changing the collective cooperation threshold *T* over time on the proportions of infected and cooperating to detect potential hysteresis effects. Figure 4 shows how the proportions of the population infected and cooperating change over time as the threshold *T* increases from 0 to 1 and then back from 1 to 0. We note that there is a dramatic difference between the forward infection and cooperation proportion curves as *T* increases from its minimum, and the corresponding curves as *T* decreases from its maximum. In particular, when *T* decreases continuously from 1 to 0, it takes longer for the proportions to begin shifting, and when they do, the decline in infection and increase in cooperation proportions occurs directly without any oscillatory behavior. See the “Methods and calculations” section for the analysis details.

## Discussion

Here we have introduced a model that generalizes the SIS model to include collective effects. The main differences from the original SIS model are that the constant transmission rate *g* is replaced by a cooperation-dependent transmission rate $g(1-C)$ and that the proportion cooperating *C* is governed by a logistic-type equation with an additional factor that governs whether the proportion cooperating is increasing or decreasing, depending on whether the proportion infected *I* is larger than a collective cooperation threshold *T*.

The general model has four equilibria whose stability properties are dependent on the three parameters *g*, *r*, and *T*. Two of these equilibria (1 and 3) correspond to the standard SIS model equilibria with the expected stability properties^21^. Specifically, the extinction equilibrium $(I,C) =(0,0)$ is stable for $g<r$ and the spread equilibrium $(I,C) = (1-\frac{r}{g},0)$ is stable for $g>r$. We note that both of these equilibria have $C=0$ so there is no cooperation. Equilibrium 2 $(I,C) =(0,1)$ where there is full cooperation and the infection vanishes is not observed in the standard SIS model, but it is also largely irrelevant here since it is always unstable. The interesting equilibrium in our model is equilibrium 4 $(I,C) = (T, 1+ \frac{r}{g(T-1)})$ where the proportion infected approaches the collective cooperation threshold as the proportion cooperating approaches $1+ \frac{r}{g(T-1)}$. Depending on the parameter values this equilibrium can be a stable spiral or a stable node. In particular, over a large range of parameter values, it is a stable spiral ($r<A$ and $g>\Gamma$) and in this case, the model generates damped oscillation in the proportion of infected. This general model may be useful as a base model for any disease spread where reinfection is common^48,49^ so an SIS model is appropriate, collective behavior affects the spread^8,9,12,18,19^, and the collective cooperation response depends on disease prevalence relative to a community-specific threshold^19,20^.

We then fixed the recovery rate to $r=1/14$ which corresponds to the typical recovery time of approximately two weeks for COVID-19^53^. The resulting phase diagram in only *g* and *T* is presented in Fig. 2 and we note the standard SIS model equilibria (1 and 3), as well as both the stable node and stable spiral forms of equilibrium 4, are still present. In particular, the stable spiral form of equilibrium 4 which corresponds to damped oscillations in the proportion of infected over time, consistent with the waves of infection observed during COVID-19^5–7^, is present over a large range of *g* and *T* parameter values, and that as the transmission rate increases, the range of collective action thresholds *T* over which oscillatory behavior (spiral) rather than a direct approach to the equilibrium (node) increases with the threshold *T*. This restricted version of the model may be particularly useful for considering the impacts of different strains of COVID-19. As mutations became favorable to the SARS-CoV-2 virus, new strains became increasingly infectious, with Delta and Omicron having higher $R_0$ values compared to earlier variants^4^, corresponding to higher *g* transmission rates. Especially because many newer variants are regional^55^, understanding the thresholds required to limit infection spread in places with unique variants could be useful for future preparation. Models investigating spread differences between distinct COVID-19 variants^56,57^ could be combined with our approach to understand the changes in the degree of collective cooperation necessary to limit disease spread for different strains.

To obtain a better understanding of the impact of the collective cooperation threshold *T* for ancestral COVID-19 strain parameters we fixed the transmission rate $g=0.2$ calculated from the reported $R_0$ value of 2.9^54^, keeping the $r=1/14$ from the previous analysis. For a given fixed *T* the model produces the long-term dynamics illustrated in Fig. 3. Specifically, for $T<0.61$, equilibrium 4 is a stable spiral, which corresponds to damped oscillations in the proportion of infected over time. For $0.61<T<0.64$, equilibrium 4 is a stable node, and for $T>0.64$ equilibrium 3 is a stable node. We note that equilibrium 3 is a standard SIS model equilibrium with no cooperation, suggesting that in the current model for ancestral COVID-19, cooperation only influences the spread if the threshold is sufficiently low. This suggests that in communities with high thresholds for cooperative behavior, even if cooperation is present, it will have no long-term effect and the disease spread will be SIS-like. Our model is able to reproduce the timing of the original spikes of COVID-19 in 2020 in the United States. At a low threshold, as shown in Fig. 3b, oscillations restart roughly every 100 days. WHO data for weekly cases in the United States indicate major spikes starting in early April, mid-July, and mid-to-late October, each around three months apart^58^. This indicates that our model, when only accounting for recovery rate, transmission rate, and a threshold on collective behavior, can replicate qualitative features of COVID-19 spread that match published data. However, the type of data-driven modeling and forecasting activities presented in studies with more complex models, e.g.^25–27,31,36,39,42,44,46,50,57,59,60^ are beyond the capabilities of our minimal model alone and the scope of our investigation.

Throughout the COVID pandemic most communities have gone through periods with stricter and more lax restrictions^11,20^, as well as changes in public perception of the danger of the disease^12–14^, and how the media reports on the disease^15–17^. Such changes would correspond to a change of *T* in our model. *T* would decrease when the individuals in a community become more concerned, and increase when a community becomes less concerned. To determine if the diagram in Fig. 3a, in particular, the location of the *T* limits, would be appropriate for determining the outcomes as the *T* parameter changes over time, we performed simple hysteresis analysis and found strong hysteresis effects (Fig. 4). In particular, we observe that when the proportion infected starts relatively high with no cooperation, even as *T* decreases, it takes much longer for cooperation to commence than it took for cooperation to cease when going in the other direction. This suggests that using short-term assessments to determine whether certain COVID-19 strategies are effective may not be appropriate, because it could take time to see the effects on a system. This finding could contribute to explaining the result seen in^11^ in which it took up to four weeks after the most stringent social distancing guidelines were put into place for daily cases and deaths to decrease in the United States, and even longer in Russia. More broadly, hysteresis effects are known to influence processes across the physical and life sciences, including infectious disease epidemiology^61^, and our work here may offer a potential explanation for some of these in terms of collective behavior.

Many available COVID-19 models are complex SIR+ models^23,25,28–36,38^ which may be disadvantageous because the explanatory power of a model tends to decrease with complexity. Especially, when complexity is added by components that may be of limited relevance, e.g. the R compartment in SIR+ models of COVID-19 since recurrent individual reinfection is frequent^48,49^. Therefore, to explain some fundamental features of COVID-19 an SIS model may be more appropriate. Furthermore, many COVID-19 models do not account for collective behavior^23–27,39–43,49,50^. While each individual has the power to choose the degree to which they follow mitigation efforts, it is the collective behavior of a community that drives spread, not a single individual, indicating why population behavior is vital to understand and model^12,18^. The model proposed here aims to fill the gap in understanding how collective behavior contributes to disease spread in its simplest form. Because it reduces to standard SIS spread when there is no cooperation, all differences between this model and the standard SIS spread are due to the incorporated collective behavior. The information provided by this model can be used to evaluate disease mitigation strategies or identify important population thresholds in preparation for future pandemics.

## Methods and calculations

### Qualitative analysis

Here we present a brief overview of the method and then apply it to obtain our main result. For a general system of nonlinear, autonomous differential equations of the form

2 $$\begin{aligned} {\left\{ \begin{array}{ll} \displaystyle {\dot{I}}=f(I,C) \\ {\dot{C}}=g(I,C) \end{array}\right. } \end{aligned}$$

the equilibria are the solutions $(I^*,C^*)$ to the system

3 $$\begin{aligned} {\left\{ \begin{array}{ll} \displaystyle f(I^*,C^*)=0 \\ g(I^*,C^*)=0 \end{array}\right. } \end{aligned}$$

The stability properties of each equilibrium $(I^*,C^*)$ is then found by calculating the eigenvalues $\lambda _1$ and $\lambda _2$ of the Jacobian matrix *J* evaluated at the equilibrium

4 $$\begin{aligned} J(I^*,C^*)=\begin{bmatrix} f_I (I^*,C^*) &{} f_C (I^*,C^*) \\ g_I (I^*,C^*) &{} g_C (I^*,C^*) \end{bmatrix} \end{aligned}$$

where subscripts indicate the partial derivative with respect to that variable. Once the eigenvalue have been obtained the Hartman-Grobman theorem^62^ can be used to determine the stability type of the equilibrium. The theorem parts that are relevant to our work are summarized here. If both eigenvalues are real and distinct, the equilibrium will be an unstable node if $0 < \lambda _1 \le \lambda _2$, a stable node if $\lambda _1 \le \lambda _2 < 0$, and a saddle if $\lambda _1< 0 < \lambda _2$. For complex eigenvalues of the form $\lambda = a \pm bi$, the equilibrium will be a stable spiral if $a < 0$ and an unstable spiral if $a > 0$.

Below we apply this method to establish our main result for the general model. Recall that we only consider *g*, *r*, *T* in (0, 1) and $g \ne r$. These conditions are used throughout.

#### Equilibria of our model

To find the equilibria of our model 1 we solve the following system of equations

5 $$\begin{aligned} {\left\{ \begin{array}{ll} \displaystyle g(1-C)I(1-I)-rI = 0 \\ C(1-C)(I-T) =0 \end{array}\right. } \end{aligned}$$

Note that $C(1-C)(I-T) = 0$ if $C=0$, $1-C = 0$, and/or $I-T = 0$.

If $C= 0$, the top equation simplifies to $I(g-gI-r) = 0$, which has two solutions $I=0$ and $I=1-\frac{r}{g}$.

If $C=1$, the top equation simplifies to $-rI = 0$ which has the solution $I = 0$.

If $I = T$, the top equation simplifies to $C = 1-\frac{r}{g(1-T)}$.

Therefore, the four equilibria of the model are

1. $$I = 0$$, $$C=0$$
2. $$I = 0$$, $$C = 1$$
3. $$I = 1-\frac{r}{g}$$, $$C = 0$$
4. $$I = T$$, $$C = 1-\frac{r}{g(1-T)}$$

#### Stability of the equilibria in our model

The Jacobian corresponding to the linearization of our model becomes

6 $$\begin{aligned} J(I,C)=\begin{bmatrix} g(2I-1)(C-1)-r &{} gI(I-1) \\ C-C^2 &{} 2TC-2IC-+I-T \end{bmatrix} \end{aligned}$$

and calculating the eigenvalues of the Jacobian evaluated at each the four equilibria $(I^*,C^*)$ gives their stability properties.

#### Equilibrium 1: (0, 0)

7 $$\begin{aligned} J(0,0)=\begin{bmatrix} g-r &{} 0 \\ 0 &{} -T \end{bmatrix} \end{aligned}$$

has the eigenvalues $\lambda _1 = g-r$ and $\lambda _2 = -T$. $\lambda _2$ is always negative. $\lambda _1 < 0$ when $g<r$, and $\lambda _1 > 0$ when $g>r$. Therefore, equilibrium 1 (0, 0) is a stable node when $g<r$, and unstable (node) when $g>r$, for all *T*.

#### Equilibrium 2: (0, 1)

8 $$\begin{aligned} J(0,1)=\begin{bmatrix} -r &{} 0 \\ 0 &{} T \end{bmatrix} \end{aligned}$$

has the eigenvalues $\lambda _1 = -r$ and $\lambda _2 = T.$ $\lambda _1$ is always negative and $\lambda _2$ is always positive. Therefore, equilibrium 2 (0, 1) is always unstable (saddle).

#### Equilibrium 3: $(1-\frac{r}{g},0)$

9 $$\begin{aligned} J(1-\frac{r}{g},0)=\begin{bmatrix} r-g &{} \frac{r^2}{g}-r \\ 0 &{} 1-\frac{r}{g}-T \end{bmatrix} \end{aligned}$$

has the eigenvalues $\lambda _1 = r-g$ and $\lambda _2 = 1-T-\frac{r}{g}$. $\lambda _1 < 0$ when $g>r$. $\lambda _2 < 0$ when $1-T-\frac{r}{g}<0$ which is equivalent to $g<\frac{r}{1-T}$. Noting that $T\in (0,1)$ implies that $r < \frac{r}{1-T}$ we can combine the two criteria into one $r<g<\frac{r}{1-T}$. Therefore, equilibrium 3 $(1-\frac{r}{g},0)$ is a stable node when $r<g<\Delta$, where $\Delta = \frac{r}{1-T}$, and unstable otherwise.

#### Equilibrium 4: $(T,1-\frac{r}{g(1-T)})$

10 $$\begin{aligned} J(T,1-\frac{r}{g(1-T)})=\begin{bmatrix} \frac{rT}{T-1} &{} gT(T-1) \\ -\frac{r(gT-g+r)}{g^2(T-1)^2} &{} 0 \end{bmatrix} \end{aligned}$$

has the eigenvalues

11 $$\begin{aligned} \lambda _{1,2} = \frac{rgT\pm \sqrt{-4((-\frac{gT}{4}+T-1)r+g(T-1)^2)rgT}}{2g(T-1)}=-\frac{T\Delta }{2} \left( 1 \pm \sqrt{\frac{(g-\Gamma )(r-A)}{gr}}\right) \end{aligned}$$

where $$A = \frac{4(T-1)^2}{T}$$, $$\Gamma = \frac{4r(1-T)}{4(T-1)^2-rT}$$, and $$\Delta = \frac{r}{1-T}.$$

These eigenvalues will be a complex conjugate pair $\lambda =a\pm bi$ (with $a=-\frac{T\Delta }{2}<0$) if and only if $(g-\Gamma )(r-A)<0$. For unconstrained parameters this results from either of the following two cases

1. $$g>\Gamma$$ and $$r<A$$.
2. $$g<\Gamma$$ and $$r>A$$.

To determine the relevant case for our constrained parameters we note that $\Gamma$ can be expressed in terms of *A* as

12 $$\begin{aligned} \Gamma =\frac{4r(1-T)}{T(A-r)}. \end{aligned}$$

Note that the numerator is always positive. Now, if $r<A$ the denominator is also positive so $\Gamma >0$. However, if $r>A$ the denominator is negative so $\Gamma <0$, and then there are no $g>0$ that satisfies the condition $g<\Gamma$ for case 2 above, and it is thus irrelevant here. Therefore, we conclude that equilibrium 4 is a stable spiral only for $g>\Gamma$ and $r<A$.

The eigenvalues will be a real if and only if $(g-\Gamma )(r-A)>0$ which for unconstrained parameters result from either of the following two cases

1. $$g>\Gamma$$ and $$r>A$$.
2. $$g<\Gamma$$ and $$r<A$$.

We are interested in when the equilibrium will be stable (node), which requires both eigenvalues to be negative. From Eq. (11) we note that this will occur when

13 $$\begin{aligned}{} & {} 1 - \sqrt{\frac{(g-\Gamma )(r-A)}{gr}}>0 \end{aligned}$$

14 $$\begin{aligned}{} & {} g>\frac{\Gamma (A-r)}{A}=\frac{r}{1-T}=\Delta . \end{aligned}$$

Including this condition ($g>\Delta$) in cases 1 and 2 above gives

1. For *g* to satisfy both $g>\Gamma$ and $g>\Delta$ we must have $g>\max \{\Gamma ,\Delta \}=\Delta$. The reason $\max \{\Gamma ,\Delta \}=\Delta$ here is because when $r>A$
  15 $$\begin{aligned} \Gamma =\frac{4r(1-T)}{T(A-r)}<0 \end{aligned}$$
  and $\Delta$ is always positive so $\Gamma<0<\Delta$.
2. For *g* to satisfy both $g<\Gamma$ and $g>\Delta$ we must have $\Delta<g<\Gamma$. We also note that $\Delta <\Gamma$ when $r>A$ so there is a range of *g* over which case 2 is relevant.

Therefore we conclude that Equilibrium 4 is a stable node when

1. $$g>\Delta$$ and $$r>A$$, and
2. $$\Delta<g<\Gamma$$ and $$r<A$$.

#### Summary stability analysis

1. (0, 0) is a stable node when $g<r$.
2. (0, 1) is always unstable.
3. $(1-\frac{r}{g},0)$ is a stable node when $r<g<\Delta$.
4. $$(T,1-\frac{r}{g(1-T)})$$ is a

stable spiral when $r<A$ and $g>\Gamma$,

a stable node if $r<A$ and $\Delta< g < \Gamma$,

a stable node if $r>A$ and $g>\Delta$.

### Hysteresis analysis

To investigate the impacts of changing the collective cooperation threshold *T* over time on the proportion of infected *I* and cooperating *C* in the model with COVID-19 recovery rate $r = 1/14$ and transmission rate $g = 0.2$ we used the Matlab function ode45 to continuously solve the system as the parameter *T* was varied. Initially, *T* was set to 0 and increased incrementally to 1 over a time period of 10,000-time steps. The values for the proportion infected and cooperating from the final increase time step were then used as initial conditions for the decrease phase where the threshold *T* decreased from 1 down to 0. See the Code availability statement for how to access the complete code needed to perform the calculations and plot the result.

## Data Availability

The code required to verify the computational part of this work can be found here https://github.com/danielstrombom/SIcollective.

## References

[1] Huremović D (2019). Psychiatry of pandemics: a mental health response to infection outbreak.

[2] Cohen, J. & Kupferschmidt, K. Labs scramble to produce new coronavirus diagnostics. *Science* **367**, (2020). 10.1126/science.367.6479.727 32054740

[3] Nature. Coronavirus: the first three months as it happened. *Nat. News* (2020). 10.1038/d41586-020-00154-w 32152592

[4] Liu Y, Rocklöv J (2022). The effective reproductive number of the Omicron variant of SARS-CoV-2 is several times relative to Delta. J. Travel Med..

[5] Zhang, S. X., Arroyo Marioli, F., Gao, R. & Wang, S. A second wave? What do people mean by COVID waves?–a working definition of epidemic waves. *Risk Manag. Healthc. Policy* 3775–3782 (2021). 10.2147/RMHP.S326051 PMC8448159 34548826

[6] Rypdal K, Bianchi FM, Rypdal M (2020). Intervention fatigue is the primary cause of strong secondary waves in the COVID-19 pandemic. Int. J. Environ. Res. Public Health.

[7] Bergman A, Sella Y, Agre P, Casadevall A (2020). Oscillations in US COVID-19 incidence and mortality data reflect diagnostic and reporting factors. Msystems.

[8] Walker PG (2020). The impact of COVID-19 and strategies for mitigation and suppression in low-and middle-income countries. Science.

[9] Lin L, Zhao Y, Chen B, He D (2022). Multiple COVID-19 waves and vaccination effectiveness in the United States. Int. J. Environ. Res. Public Health.

[10] Andrejko KL (2022). Effectiveness of face mask or respirator use in indoor public settings for prevention of SARS-CoV-2 infection-California, February-December 2021. Morb. Mortal. Wkly Rep..

[11] Thu TPB, Ngoc PNH, Hai NM (2020). Effect of the social distancing measures on the spread of COVID-19 in 10 highly infected countries. Sci. Total Environ..

[12] Wise T, Zbozinek TD, Michelini G, Hagan CC, Mobbs D (2020). Changes in risk perception and self-reported protective behaviour during the first week of the COVID-19 pandemic in the United States. R. Soc. Open Sci..

[13] Chen W, Stoecker C (2020). Mass media coverage and influenza vaccine uptake. Vaccine.

[14] Brug J (2004). SARS risk perception, knowledge, precautions, and information sources, the Netherlands. Emerg. Infect. Dis..

[15] Oh S-H, Lee SY, Han C (2021). The effects of social media use on preventive behaviors during infectious disease outbreaks: The mediating role of self-relevant emotions and public risk perception. Health Commun..

[16] Melki J (2022). Media exposure and health behavior during pandemics: The mediating effect of perceived knowledge and fear on compliance with COVID-19 prevention measures. Health Commun..

[17] Fridman I, Lucas N, Henke D, Zigler CK (2020). Association between public knowledge about COVID-19, trust in information sources, and adherence to social distancing: Cross-sectional survey. JMIR Public Health Surveill..

[18] Chen, J. *et al.* Individual and collective behavior in public health epidemiology. In *Handbook of statistics*, vol. 36, 329–365 (Elsevier, 2017).

[19] Caley P, Philp D, McCracken K (2008). Quantifying social distancing arising from pandemic influenza. JR Soc Interface..

[20] Singh S, Shaikh M, Hauck K, Miraldo M (2021). Impacts of introducing and lifting nonpharmaceutical interventions on COVID-19 daily growth rate and compliance in the United States. Proc. Natl. Acad. Sci..

[21] Otto SP, Day T (2007). A biologist’s guide to mathematical modeling in ecology and evolution.

[22] Meredith HR (2021). Coordinated strategy for a model-based decision support tool for coronavirus disease, Utah, USA. Emerg. Infect. Dis..

[23] Leontitsis A (2021). Seahir: A specialized compartmental model for covid-19. Int. J. Environ. Res. Public Health.

[24] Chen Y-C, Lu P-E, Chang C-S, Liu T-H (2020). A time-dependent SIR model for COVID-19 with undetectable infected persons. IEEE Trans. Netw. Sci. Eng..

[25] Batistela CM, Correa DP, Bueno ÁM, Piqueira JRC (2021). SIRSi compartmental model for COVID-19 pandemic with immunity loss. Chaos Solitons Fract..

[26] Dashtbali M, Mirzaie M (2021). A compartmental model that predicts the effect of social distancing and vaccination on controlling COVID-19. Sci. Rep..

[27] Ramezani SB, Amirlatifi A, Rahimi S (2021). A novel compartmental model to capture the nonlinear trend of COVID-19. Comput. Biol. Med..

[28] He S, Peng Y, Sun K (2020). SEIR modeling of the COVID-19 and its dynamics. Nonlinear Dyn..

[29] Annas S, Pratama MI, Rifandi M, Sanusi W, Side S (2020). Stability analysis and numerical simulation of SEIR model for pandemic COVID-19 spread in Indonesia. Chaos Solitons Fract..

[30] Mwalili S, Kimathi M, Ojiambo V, Gathungu D, Mbogo R (2020). SEIR model for COVID-19 dynamics incorporating the environment and social distancing. BMC. Res. Notes.

[31] Samui P, Mondal J, Khajanchi S (2020). A mathematical model for COVID-19 transmission dynamics with a case study of India. Chaos Solitons Fract..

[32] Gevertz JL, Greene JM, Sanchez-Tapia CH, Sontag ED (2021). A novel COVID-19 epidemiological model with explicit susceptible and asymptomatic isolation compartments reveals unexpected consequences of timing social distancing. J. Theor. Biol..

[33] Raimúndez E (2021). COVID-19 outbreak in Wuhan demonstrates the limitations of publicly available case numbers for epidemiological modeling. Epidemics.

[34] Arino J, Portet S (2020). A simple model for COVID-19. Infect. Dis. Model..

[35] Chen X (2021). Age-stratified COVID-19 spread analysis and vaccination: A multitype random network approach. IEEE Trans. Netw. Sci. Eng..

[36] Giordano G (2020). Modelling the COVID-19 epidemic and implementation of population-wide interventions in Italy. Nat. Med..

[37] Anderson RM (1991). Discussion: The Kermack-McKendrick epidemic threshold theorem. Bull. Math. Biol..

[38] Yang Z (2020). Modified SEIR and AI prediction of the epidemics trend of COVID-19 in China under public health interventions. J. Thorac. Dis..

[39] Kerr CC (2021). Covasim: An agent-based model of COVID-19 dynamics and interventions. PLoS Comput. Biol..

[40] Cuevas E (2020). An agent-based model to evaluate the COVID-19 transmission risks in facilities. Comput. Biol. Med..

[41] Wolfram C (2020). An agent-based model of covid-19. Complex Syst..

[42] Shamil, M. S., Farheen, F., Ibtehaz, N., Khan, I. M. & Rahman, M. S. An agent-based modeling of COVID-19: validation, analysis, and recommendations. *Cogn. Computa.* 1–12 (2021). 10.1007/s12559-020-09801-w PMC7893846 33643473

[43] Silva PC (2020). COVID-ABS: An agent-based model of COVID-19 epidemic to simulate health and economic effects of social distancing interventions. Chaos Solitons Fract..

[44] Palomo-Briones GA, Siller M, Grignard A (2022). An agent-based model of the dual causality between individual and collective behaviors in an epidemic. Comput. Biol. Med..

[45] Bittihn P, Hupe L, Isensee J, Golestanian R (2021). Local measures enable COVID-19 containment with fewer restrictions due to cooperative effects. EClinicalMedicine.

[46] Engle S (2021). The behavioral SIR model, with applications to the swine flu and COVID-19 pandemics.

[47] Wan J (2022). Multilayer networks with higher-order interaction reveal the impact of collective behavior on epidemic dynamics. Chaos Solitons Fract..

[48] Mei Q (2020). Assessment of patients who tested positive for COVID-19 after recovery. Lancet. Infect. Dis.

[49] Kosmidis K, Macheras P (2020). A fractal kinetics SI model can explain the dynamics of COVID-19 epidemics. PLoS ONE.

[50] Demongeot J, Griette Q, Magal P (2020). SI epidemic model applied to COVID-19 data in mainland China. R. Soc. Open Sci..

[51] Aplin, L.M, Farine, D. R., Morand-Ferron, J., Cockburn, A., Thornton, A., & Sheldon, B. C. Experimentally induced innovations lead to persistent culture via conformity in wild birds. *Nature* **518**, 538–541 (2015). 10.1038/nature13998 PMC4344839 25470065

[52] Rogers, E. M. *Diffusion of Innovations, Fifth Edition* (Simon & Schuster, Inc., New York, NY, USA., 2003).

[53] Backer JA, Klinkenberg D, Wallinga J (2020). Incubation period of 2019 novel coronavirus (2019-nCoV) infections among travellers from Wuhan, China, 20–28 January 2020. Eurosurveillance.

[54] Billah MA, Miah MM, Khan MN (2020). Reproductive number of coronavirus: A systematic review and meta-analysis based on global level evidence. PLoS ONE.

[55] Zhou D (2021). Evidence of escape of SARS-CoV-2 variant B. 1.351 from natural and vaccine-induced sera. Cell.

[56] Zhao Y (2022). The global transmission of new coronavirus variants. Environ. Res..

[57] LaJoie Z, Usherwood T, Sampath S, Srivastava V (2022). A COVID-19 model incorporating variants, vaccination, waning immunity, and population behavior. Sci. Rep..

[58] World Health Organization. WHO Coronavirus (COVID-19) Dashboard.

[59] He, R., Luo, X., Asamoah, J.K.K., Zhang, Y., Li, Y., Jin, Z., & Sun, G.-Q. A hierarchical intervention scheme based on epidemic severity in a community network. *J. Math. Biol.* **87**, 29 (2023). 10.1007/s00285-023-01964-y 37452969

[60] Luo, X.-F., Feng, S., Yang, J., Peng, X.-L., Cao, X., Zhang, J., Yao, M., Zhu, H., Li, M. Y. Wang, H., et al. Nonpharmaceutical interventions contribute to the control of COVID-19 in China based on a pairwise model. *Infect. Dis. Model.* **6**, 643–663 (2021). 10.1016/j.idm.2021.04.001 PMC8035808 33869909

[61] Lacitignola D, Saccomandi G (2021). Managing awareness can avoid hysteresis in disease spread: an application to coronavirus Covid-19. Chaos Solitons Fract..

[62] Hartman P (1960). A lemma in the theory of structural stability of differential equations. Proc. Am. Math. Soc..

